# Tetanus Cured by Massive Doses of Serum

**Published:** 1916-10

**Authors:** 


**Tetanus Cured by Massive Doses of Serum.** Gras, Le Progres Med., Aug. 5, reports the case of a cavalry soldier who received several small scalp wounds from being struck by the head of a horse. 12 days later he developed acute tetanus. 6 injections of 100 c.c. each were given in 15 days with complete recovery.

